# Modulation of Spliceosomal Proteins hnRNPH1 and H2 Increases Melanoma Cell Pro-Inflammatory Signaling In Vitro

**DOI:** 10.3390/biom15111611

**Published:** 2025-11-17

**Authors:** Maab Sultan, Shuai Ma, Juan Diez, Sadeeshkumar Velayutham, Yousef Al-Harbi, Jun Yong Choi, Keiran S. M. Smalley, Lubov Nathanson, Vladimir Beljanski, Dmitriy Minond

**Affiliations:** 1 Barry and Judy Silverman College of Pharmacy, Nova Southeastern University, 3321 College Avenue, Fort Lauderdale, FL 33314, USAvsadeesh@nova.edu (S.V.);; 2 Ph.D. Program in Chemistry, The Graduate Center of the City University of New York, New York, NY 10016, USA; 3 Rumbaugh-Goodwin Institute for Cancer Research, Nova Southeastern University, 3321 College Avenue, CCR r.605, Fort Lauderdale, FL 33314, USA; 4 Department of Pharmacology and Toxicology, College of Pharmacy, Qassim University, Buraidah 52571, Saudi Arabia; 5 Department of Chemistry and Biochemistry, Queens College, 65-30 Kissena Boulevard, Flushing, NY 11367, USA; 6 Department of Tumor Biology, Moffitt Cancer Center, 12902 Magnolia Drive, Tampa, FL 33612, USA; 7 Dr. Kiran C. Patel College of Osteopathic Medicine, Nova Southeastern University, 3321 College Avenue, Fort Lauderdale, FL 33314, USA; lnathanson@nova.edu; 8 Dr. Kiran C. Patel College of Allopathic Medicine, Nova Southeastern University, 3321 College Avenue, Fort Lauderdale, FL 33314, USA

**Keywords:** melanoma, spliceosomal proteins, hnRNPH1/H2, drug discovery, NanoString, immunotherapy

## Abstract

Melanoma is the most aggressive and deadliest form of skin cancer, and the current treatments of melanoma have many limitations, which necessitate discovering new compounds and targets for melanoma. Two probes, 2155-14 and 2155-18, were identified to induce apoptotic cell death, autophagy, and immune signaling modulation through hnRNPH1/H2-dependent mechanisms. RNA sequencing following the siRNA-mediated knockdown of hnRNPH2 in melanoma cells revealed an enrichment of immune-related signaling pathways. The present study investigated the effect of genetic and pharmacologic downregulation of hnRNPH1/H2 on melanoma immunogenicity in vitro. Our results indicated that treating melanoma cell lines with 2155-14 and 2155-18 led to hnRNPH1/H2 downregulation, whereas hnRNPH2 siRNA treatment led to only hnRNPH2 downregulation. Both types of treatment resulted in a significant upregulation of pro-inflammatory pathways and simultaneous downregulation of anti-inflammatory pathways. These findings provide the first insight into the role of hnRNPH1/H2 as critical drivers of melanoma immunogenicity and suggest their potential as novel therapeutic targets for enhancing melanoma treatment outcomes. This study underscores the impact of post-transcriptional regulation on the immune environment in melanoma and in cancer in general.

## 1. Introduction

Melanoma is a highly aggressive and one of the deadliest forms of skin cancer. In 2022, an estimated 331,722 new cases of melanoma were diagnosed globally, and mortality due to melanoma accounted for 58,667 deaths. North America ranks second in the highest incidence and prevalence of melanoma cases, following Europe [1]. The average annual cost of melanoma treatment in the United States was approximately $2.5 billion between 2016 and 2018 [2]. Current treatments for melanoma face significant limitations, including low response rates (RRs), a lack of effective therapies for triple-wild-type and NRAS-mutant melanoma [3], and drug resistance [4,5].

We previously discovered two small-molecule probes, 2155-14 and 2155-18, which showed potency against melanoma cell lines that was comparable to vemurafenib [6]. Pull-down assays using lysates of melanoma cell lines M14 and WM266-4 with biotinylated analogs of 2155-14 revealed four specific bands. The four bands were identified by LC/MS/MS as ATP-dependent RNA helicase DDX1 (band 1), heterogeneous nuclear ribonucleoprotein H2 (hnRNPH2, band 2), and heterogeneous nuclear ribonucleoprotein A2/B1 (hnRNPA2/B1, bands 3 and 4), all of which were confirmed by immunoblotting [7]. siRNA-mediated knockdown of these targets revealed that only the knockdown of hnRNPH2 potentiates autophagy and apoptosis. Due to the 96% sequence homology of hnRNPH1 and hnRNPH2 sequences, a high degree of functional similarity between the two proteins was suggested [8]. Both hnRNPH1 and hnRNPH2 are RNA-binding proteins (RBPs) that play a key role in regulating essential RNA-related transcription processes. Members of the hnRNP family regulate or contribute to the regulation of splicing, transcription, mRNA trafficking, polyadenylation, telomere maintenance, translational regulation, and mRNA degradation [9,10].

Targeting RNA-binding proteins to modulate splicing in cancer cells is a relatively new approach in cancer therapy [11,12]. Nevertheless, two compounds—E7107 and H3B-8800—that target the spliceosomal protein, SF3B1, have shown anticancer effects in preclinical and clinical studies. A Phase I clinical study of E7107 in patients with advanced solid tumors was terminated due to adverse events (clinical trial # NCT00499499). H3B-8800 has been evaluated in clinical settings for patients with myelodysplastic syndromes and leukemia (clinical trial #NCT02841540). The results demonstrate dose-dependent target engagement and safety of H3B-8800 even with prolonged dosing [13].

A previous in vivo study demonstrated that the administration of BRAF inhibitors to mice carrying melanoma tumors following anti-PD-1 therapy resulted in increased infiltration of monocytes, T cells, natural killer cells, and dendritic cells, alongside a reduction in Tregs, myeloid-derived suppressor cells (MDSCs), and tumor-associated macrophages [14]. Such results suggest that pharmacological modulation of an “unrelated” target can enhance immune responses against tumor cells. Furthermore, a recent clinical informatics study using data from the Cancer Genome Atlas (TCGA) found a correlation between the increased expression of immune-related signaling pathways (interferon-α/β and interferon-γ) and improved overall survival in melanoma patients [15]. This finding is significant due to the association of interferon-α/β and interferon-γ pathways with higher immune cell infiltration into tumors [16,17], which results in a better response to immunotherapy.

In the present study, we demonstrate the effect of pharmacological and siRNA-mediated downregulation of hnRNPH2 on stimulation of pro-inflammatory signaling in melanoma cells.

## 2. Materials and Methods

**Procedure for the synthesis of 2155-14 (JC-395) and 2155-18 (JC-408).** Pyrrolidine–bis-diketopiperazine JC-395 and JC-408 (Figure S1) were synthesized by modifying the previously published method [6]. The key intermediate (**5**) was synthesized using automated microwave synthesis conditions on a CEM Liberty PRIME 2.0 system with the one-pot coupling/deprotection methodology. Couplings were performed with Fmoc-protected amino acid (0.5 M in DMF), DIC (1.0 M in DMF), and Oxyma (0.3 M in DMF) for 30 s at 25 °C, followed by 4 min at 90 °C. Fmoc deprotection was performed for 2 min at 110 °C and initiated by adding pyrrolidine/DMF (15% *v*/*v*) directly to the undrained post-coupling solution. The mBHA resin (1.42 g, 2 mmol) was washed with 20% piperidine/DMF (6 mL, 3 times), and it was transferred to the 125 mL reaction vessel for the microwave-assisted amide coupling and Fmoc deprotection reactions. The deprotection-coupling cycle runs were automatically performed with Fmoc-protected amino acids and carboxylic acid. Fmoc-L-Phe, Fmoc-L-Pro, Fmoc-cyclohexylalanine, Fmoc-L-Tyr(OtBu), and phenylacetic acid were sequentially used for the intermediate (**5**) of 2155-14 (JC-395), while Fmoc-L-Phe, Fmoc-L-Pro, Fmoc-L-Phe, Fmoc-L-Tyr(OtBu), and admantylacetic acid were used for the synthesis of 2155-18 (JC-408).

After coupling reactions, the reaction solution was poured off into a disposable 24 mL polypropylene syringe fitted with a PTFE filter. The solution was drained from the syringe, and the resin was washed with DMF (6 × 6 mL) and MeOH (6 × 6 mL) and allowed to air-dry. The amide groups on the air-dry resin were reduced using a 40× excess of borane (1 M in tetrahydrofuran (THF)) in the round-bottomed flask under nitrogen at 65 °C for 72 h. The solution mixture was quenched with methanol (MeOH, 20 mL), and the resin was washed with THF (6 × 6 mL) and MeOH (6 × 6 mL) and allowed to air-dry. Then the resin was treated with piperidine (20 mL), which was stirred at 65 °C for 18 h in a glass vessel. The solution was poured off and drained from the syringe. The resin was washed with DMF (6 × 6 mL) followed by MeOH (6 × 6 mL) and allowed to air-dry. Completion of reduction was monitored by cleaving 10–15 beads of the resin and analyzing the cleaved product mixture using LC and MS. The resin was added to a solution of 1,1′-oxalyldiimidazole (10 equiv.) in amine-free anhydrous DMF (0.1 M) and shaken at 25 °C for 20 h. The solution was poured off and drained from the syringe, and the resin was rinsed with DMF (6 × 6 mL) and MeOH (6 × 6 mL). Completion of cyclization was monitored by cleaving 10–15 beads of resin and analyzing the product mixture by LC and MS. The resin was treated with a cleavage solution (12 mL) of trifluoroacetic acid (TFA)/trifluoromethanesulfonic acid (TFMSA) (9:1) for 18 h at 25 °C. After the reaction was completed, the cleavage solution was collected, and TFA was removed under a nitrogen blow. Saturated sodium bicarbonate solution (NaHCO_3_) was added, and the mixture was extracted with ethyl acetate (2 × 50 mL). The combined organic layers were washed with brine (30 mL), dried over anhydrous magnesium sulfate (MgSO_4_), and evaporated under the rotary evaporator. Final crude products were purified using preparative HPLC as described below to produce the title compound (JC-395 or JC-408) as a white powdery solid with >97% purity.

*(S)-1-((R)-1-((S)-2-(((S)-6-benzyl-2,3-dioxopiperazin-1-yl)methyl)pyrrolidin-1-yl)-3-cyclohexylpropan-2-yl)-5-(4-hydroxybenzyl)-4-phenethylpiperazine-2,3-dione TFA salt.*

^1^H NMR (500 MHz, DMSO-*d*_6_) δ ppm 0.93 (q, *J* = 12.2, 13.1 Hz, 2H), 1.22 (ddt, *J* = 7.6, 14.9, 40.3 Hz, 5H), 1.32–1.43 (m, 1H), 1.54–1.72 (m, 4H), 1.77 (dd, *J* = 7.9, 16.1 Hz, 2H), 1.99 (pent, *J* = 7.5, 8.0 Hz, 2H), 2.29 (dq, *J* = 6.6, 13.3 Hz, 1H), 2.73 (tt, *J* = 8.0, 13.9 Hz, 2H), 2.86 (dtd, *J* = 5.5, 12.6, 14.9, 25.4 Hz, 4H), 2.99 (td, *J* = 5.6, 14.0 Hz, 2H), 3.20 (dt, *J* = 13.2, 21.9 Hz, 3H), 3.37–3.80 (m, 10H), 3.85 (q, *J* = 6.2 Hz, 1H), 4.01 (dd, *J* = 4.8, 13.9 Hz, 1H), 4.87 (d, *J* = 10.2 Hz, 1H), 6.72 (d, *J* = 8.3 Hz, 2H), 7.03 (d, *J* = 8.1 Hz, 2H), 7.12–7.45 (m, 10H), 8.64 (d, *J* = 5.3 Hz, 1H), 9.18 (s, 1H), 9.38 (s, 1H). ^13^C NMR (126 MHz, DMSO-*d*_6_) δ ppm 20.94, 25.60, 25.93, 28.05, 32.47, 33.33, 33.48, 36.50, 36.69, 37.00, 40.78, 46.14, 46.52, 48.18, 52.73, 53.80, 55.02, 58.12, 66.30, 115.33, 115.86, 118.23, 126.37, 126.77, 126.94, 128.45, 128.59, 129.29, 129.87, 137.01, 138.84, 155.83, 156.16, 156.86, 157.56, 158.10. *m*/*z* calculated for C_44_H_56_N_5_O_5_ [M+H]^+^: 734.4276, observed: 734.4271; *m*/*z* calculated for C_44_H_55_N_5_O_5_Na [M+Na]^+^: 756.4096, observed: 756.4095.

***Compound purification and characterization*.** The final compounds were purified using preparative HPLC with a dual pump Shimadzu LC-20AP system equipped with a SunFire C18 preparative column (19 × 250 mm, 10 micron) at λ = 220 nm, with a mobile phase of (A) H_2_O (0.1% TFA)/(B) methanol (MeOH)/acetonitrile (ACN) (1:1), at a flow rate of 60 mL/min with 10% (B) for 30 s, a gradient up to 90% (B) for 9.5 min, and 90% (B) for 3 min. ^1^H NMR and ^13^C NMR spectra were recorded in DMSO-*d*_6_ on a Bruker Ascend 500 MHz spectrometer (Bruker BioSpin GmbH, Rheinstetten, Germany) at 500.13 and 125.77 MHz, respectively, and mass spectra were recorded using an Advion Mass Express (Advion, Inc., Ithaca, New York, USA). The purities of the synthesized compounds were confirmed to be greater than 97% by liquid chromatography on a Shimadzu LC-20AD instrument with SPD-20A (Shimadzu Corporation, Kyoto, Japan). The mobile phase of (A) H_2_O (0.1% formic acid)/(B) I (0.1% formic acid) was used with a gradient of 5–95% over 7 min, followed by 3 min rinse and 3 min equilibration.

**Cell Lines and Culture.** Primary adult melanocytes, murine melanoma B16-F10, and human melanoma cell lines A375 (BRAF^V600E^, female, primary) were purchased from ATCC; WM266-4 (BRAF^V600D^, female, lymph-node metastases) and WM1366 (NRAS^Q61L^, male, primary) were purchased from Rockland Immunochemicals (Limerick, PA, USA). The human melanoma cell line WM3918 (triple-wild-type, male, metastatic) [18] was generously provided by Dr. Keiran S. Smalley (Moffitt Cancer Center, Tampa, FL, USA). Murine melanoma (B16-F10) and human BRAF-mutated cell lines (A375 and WM266-4) were cultured in DMEM (Dulbecco’s Modified Eagle Medium), whereas WM1366 and WM3918 were cultured in RPMI-1640 (Roswell Park Memorial Institute), both obtained from Cytiva (Logan, UT, USA). The media was supplemented with 1% L-glutamine (Quality Biological, Gaithersburg, MD, USA), 1% penicillin–streptomycin solution (Corning, Manassas, VA, USA), and 5-10% fetal bovine serum (FBS) (StemXVivo, Minneapolis, MN, USA). Plasmocin prophylactic (InvivoGen, San Diego, CA, USA) was added when preparing the complete media to prevent mycoplasma contamination. Additionally, the media was filtered using a 0.1 µM filter to ensure sterility. All cells were maintained in a humidified incubator at 37 °C with 95% air and 5% CO_2_. Once confluency reached 70–80%, cells were passaged using TrypLE (Corning, Manassas, VA, USA) into T-25, T-75, or T-175 flasks, depending on the growth rate and the required number of cells for future experiments. A complete culture medium was used with all cell lines.

**RNA sequencing (RNAseq).** Primary adult melanocytes and the WM266-4 human melanoma cell line were treated with 50 nM of hnRNPH2 siRNA for 24 h, after which RNA was extracted using RNAzol (Molecular Research Center) according to manufacturer instructions. 500 ng of total RNA was submitted to the NSU Genomic Core, and 50-base pair single-end sequencing was performed using Illumina NextSeq500. All procedures were performed according to the manufacturer’s instructions. Quality control assessment was performed using Illumina RNAseq pipeline to estimate genomic coverage, percent alignment and nucleotide quality. Raw reads were mapped to the reference human genome (the most recent build GRCh38) using HISAT2 2.2.1 and STAR 2.7.11a software. Reads for each gene aligned by HISAT2 were counted using HTSeq software. Alignment by STAR was run with the option “quantMode TranscriptomeSAM” that allowed counting of reads aligned to each gene. Raw counts from HTSeq and STAR were imported into Bioconductor/R package DESeq2 3.22, normalized and tested for differential gene expression. This analysis was performed separately for the files produced by each aligner. In each analysis, we selected genes that were expressed differentially based on the criteria of False Discovery Rate (FDR) less than 10% and Fold Change more than 1.5 in either direction. Genes that showed differential expression after analysis of the files from both aligners were selected for further analysis. A list of these genes was imported into the STRING database for pathway analysis.

**Western blotting for hnRNP H1 and hnRNP H2.** A375, WM-266-4, and melanocyte cells were sonicated in RIPA lysis buffer containing protease inhibitors; the protein isolates were subjected to SDS-PAGE followed by transfer to a nitrocellulose membrane. hnRNP H2 was detected using a rabbit polyclonal antibody (Abgent #: AP13497b; 1:3000, in 2% milk overnight), and hnRNP H1 was detected using a rabbit monoclonal antibody (abcam EPR25302-12). After washing with TBST, the membranes were treated with chemiluminescent horseradish peroxidase detection reagent (Thermo Scientific, Cat# 32209) and exposed to autoradiography film (Denville Scientific, Inc., Metuchen, NJ, USA, cat# E3018). ImageJ software (NIH, Bethesda, MD, USA) was used to quantify the intensity of protein bands. The protein bands were normalized against loading controls (GAPDH and Actin).

**Cell Viability Assay.** The CellTiter-Glo (CTG) viability assay (Promega, Madison, WI, USA) was used to determine the half-maximal inhibitory concentration (IC_50_) values of compounds 2155-14 and 2155-18 against multiple cell lines. When cell confluency reached 50–80%, cells were harvested and plated in a 384-well assay plate. A total of 8 µL of cell suspension (1000 cells/well) was dispensed into columns 2–12, while 8 µL of media was dispensed in the first column and row and the last column and row. The plate was covered with a metal lid and incubated at 37 °C under 5% CO_2_ and 95% air for 24 h. On the second day, a dose–response curve of the test compounds was prepared immediately before adding to the cells by adding 30 µL of media into columns 1, 2, and 4–13 of a 384 polypropylene source plate. A 300 µM compound stock solution (2155-14/2155-18) was prepared from an initial 10 mM stock. From this compound stock (14/18), 45 µL was loaded into column 3, and a serial dilution was performed by transferring 15 µL from column 3 into column 4, mixing thoroughly by pipetting up and down three times. This process was repeated sequentially across columns 4 to 12. Finally, 15 µL was discarded from the last well to maintain consistent volumes. Next, 4 µL of each compound dilution or control media was transferred from the polypropylene source plate to the corresponding wells in the assay plate using a multi-channel pipettor. The assay plate was then incubated for 72 h. After incubation, 4 µL of CTG 2.0 reagent was added to each well containing medium or cell suspension. Then, cells were incubated for at least 15 min at RT in the dark. Luminescence was measured using a microplate reader (BioTek, Winooski, VT, USA). The following formula was used to calculate the percentage of cell viability:

Cell viability (%) = (100 × (raw luminescence − 0% viable control)/(100% viable control − 0% viable control)

The dose–response curve was created using GraphPad Prism 10.1 using four-parameter flexible slope non-linear regression analysis.

**Cell Treatment with 2155-14 and 2155-18 for nCounter Analysis.** Two melanoma cell lines, A375 and WM1366, were treated with 2155-14 and 2155-18 in T-25 flasks for 24 h. The treatment concentration of each compound was determined based on its IC_50_ value in the viability assay for the respective cell line. For the A375 cell line, 17 µM of 2155-14 and 30 µM of 2155-18. For the WM1366 cell line, 16 µM of 2155-14 and 77 µM of 2155-18. Following treatment, RNA extraction was performed for further downstream assays.

**siRNA Transfection for nCounter Assays.** For genetic modulation, human cell lines were utilized, including A375 and WM266-4 (BRAF-mutated), WM1366 (NRAS mutated), and WM3918 (TWT). Each cell line was seeded in two T-25 flasks using antibiotic-free regular growth media (5 mL) supplemented with FBS. Cells were incubated for 18–24 h until reaching 60–80% confluency, as Lipofectamine RNAiMax reagent is most effective at this confluency. On the second day, the growth media in both T-25 flasks were discarded and slowly replaced at the edge of the well with 5 mL of Opti-MEM medium (Thermo Fisher Scientific, Waltham, MA, USA). The flasks were then incubated for 1 h. During the incubation period, transfection reagents were prepared. The lyophilized hnRNPH2 siRNA duplex and control siRNA-H (Scr) (Santa Cruz Biotechnology, Dallas, TX, USA) were resuspended in 330 µL of RNase-free water to yield a 10 µM concentration, followed by vortexing and spinning down for 10 s. Solution A was prepared by diluting 27.58 µL of hnRNPH2 siRNA/Scr (final concentration: 50 nM) into 250 µL of Opti-MEM medium. Solution B was prepared by diluting 17 µL of Lipofectamine RNAiMax reagent (Thermo Fisher Scientific, Waltham, MA, USA) into 250 µL of Opti-MEM medium (briefly spun before use). Solution A was added directly into solution B to dilute the transfection reagent. The solution was mixed gently by pipetting up and down, followed by vortexing and incubation for 30 min at RT on an orbital shaker to allow the formation of the siRNA–lipofectamine complexes. Next, the complexes were added to the cells: hnRNPH2 siRNA–lipofectamine complex to one flask and control siRNA–lipofectamine complex to the second flask. Cells were incubated for 24 h, harvested, and viability was measured using trypan blue assay on TC20 Cell Counter (Bio-Rad), followed by RNA extraction.

**RNA Extraction for nCounter Assays.** For total RNA extraction, the miRNAeasy Mini kit was used (Qiagen, Germantown, MD, USA). After 24 h of cell transfection, the culture medium was discarded, and the cells were washed with cold phosphate-buffered saline (PBS) without calcium and magnesium (Corning, Manassas, VA, USA). Then, 700 µL of QIAzol lysis reagent was added to the flask. The adherent cells were then scraped using a cell scraper, and the resulting cell lysates were collected into Eppendorf tubes. The cell lysate was disrupted and homogenized using a 20 G needle for 15 s, followed by incubation at RT for 5 min. Next, 140 µL of chloroform (Thermo Fisher Scientific, Waltham, MA, USA) was added, mixed thoroughly to form a soapy solution, and incubated for 2–3 min at RT. Then, the samples were centrifuged at 12,000× *g* for 15 min at 4 °C. While centrifugation was ongoing, DNase I treatment was prepared by mixing 10 µL of DNase I (RNase-free) with 70 µL of RDD buffer (Qiagen, Germantown, MD, USA). Following centrifugation, the upper aqueous phase was transferred to a new collection tube, and 1.5 times the volume of 100% ethanol was added. The mixture was thoroughly mixed by pipetting. Then, 700 µL of the sample was transferred to the RNeasy Mini column in a 2 mL collection tube and centrifuged at 12,000× *g* for 30 s at RT. The flow-through was discarded, and the remaining sample was processed using the same step. Next, 350 µL of buffer RWT was added to the RNeasy Mini column, followed by centrifugation at 12,000× *g* for 30 s at RT. The flow-through was discarded. Prepared DNase was then added and incubated at RT for 15 min. Following DNase treatment, 350 µL of buffer RWT was added to the RNeasy Mini column, followed by centrifugation under the same conditions, and the flow-through was discarded. Subsequently, 500 µL of buffer RPE was added to the column and centrifuged under the same conditions, and the flow-through was discarded. This step was repeated using an additional 500 µL buffer RPE, but this time, the sample was centrifuged for 2 min, and the flow-through was discarded. Finally, the RNeasy Mini column was transferred to a new collection tube, and 33 µL of RNase-free water was added directly to the membrane. The column was centrifuged for 1 min at 12,000× *g* at RT to elute RNA. The RNA concentration was assessed using a Nanodrop (Thermo Fisher Scientific, Waltham, MA, USA). RNA samples were stored frozen at −80 °C.

**NanoString Gene Expression.** RNA samples were sent to the genomic core at either the NSU Center for Collaborative Research (CCR) or the Moffitt Cancer Center, where Qubit, TapeStation, and nCounter assays were performed, yielding high-quality RIN scores (~10). 100 ng of total RNA from each sample was used to hybridize with the nCounter Elements TagSet at 67 °C for 16 h. The TagSet consists of a reporter tag and a capture tag that hybridize to the user-designed gene-specific probe A and probe B complex. After hybridization, the samples were washed and immobilized to a cartridge using the NanoString nCounter Prep Station. Cartridges were scanned in the nCounter Digital Analyzer at 280 fields of view for the high level of sensitivity. Positive NanoString spike-in controls and 5 highly invariant genes (SAR1B, YWHAB, ETFA, SPEN and SEC24C) served as internal controls for normalization between samples. The host response panel (NanoString cat# XT-HHR-12), covering 785 genes across more than 50 annotated pathways related to host response functions, was used. This panel was used for transcriptomic analysis of hnRNPH2 siRNA-treated cells (A375, WM266-4, WM1366, and WM3918) and compound-treated cells (A375 and WM1366). Data were analyzed using three software platforms: nSolver 4.0 and Rosalind v3.16 (NanoString, Seattle, WA, USA) for gene expression interpretation and STRING for network analysis. The cutoff for differentially expressed genes (DEGs) was set to a fold change >1.4 for upregulated genes and <−1.4 for downregulated genes, with a *p*-value < 0.05. To assess specific pathway upregulation or downregulation, a proprietary Directed Global Significance Score (DGSS) was used. DGSS incorporates direction (up- or downregulated) and magnitude of gene expression changes and is positive for activated pathways or negative for repressed pathways. Comparative magnitude of pathway changes allows pathways to be prioritized for downstream pathway analysis by orthogonal means, such as Western blot.

**Statistical Analysis.** Results are expressed as mean ± SD for each experimental group, with at least three replicates per condition. Statistical comparisons between two means were conducted using an unpaired Student’s *t*-test, while comparisons involving more than two groups were performed using ANOVA to assess significant differences among treatment groups. Data were presented as a fold change in treatment groups compared to the control group. Statistical analyses were carried out using GraphPad Prism 10.1 (San Diego, CA, USA), nSolver Analysis Software 4.0 (NanoString, Seattle, WA, USA), and Rosalind v3.16 (San Diego, CA, USA), with statistical significance set at *p* < 0.05. Gene expression data were normalized based on the expression levels of housekeeping genes. The average expression levels from replicate probes of the same gene were used to determine gene-level expression.

## 3. Results

**Scheme 1:**
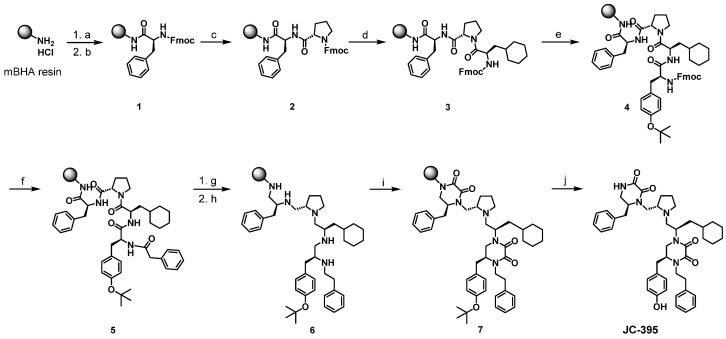
Schemes for the synthesis of 2155-14 (JC-395). Reagents and Conditions: (a) 20% piperidine/DMF, 25 °C, 3 × 30 min; (b) piperidine/DMF, 90 °C, 4 min, then Fmoc-L-Phe-OH/DIC/Oxyma, 90 °C, 4 min; (c) piperidine/DMF, 90 °C, 4 min, then Fmoc-L-Pro-OH/DIC/Oxyma, 90 °C, 4 min; (d) piperidine/DMF, 90 °C, 4 min, then Fmoc-cyclohexylalanine-OH/DIC/Oxyma, 90 °C, 4 min; (e) piperidine/DMF, 90 °C, 4 min, then Fmoc-L-Tyr(OtBu)-OH/DIC/Oxyma, 90 °C, 4 min; (f) piperidine/DMF, 90 °C, 4 min, then phenylacetic acid/DIC/Oxyma, 90 °C, 4 min; (g) Borane-THF, 65 °C, 72 h; (h) piperidine, 65 °C, 18 h; (i) 1,1′-oxalyldiimidazole (10 equiv.), free amine anhydrous DMF, 25 °C, 20 h; (j) TFA/TFMSA (9:1), 25 °C, 18 h. Synthetic schemes for JC-395 are presented as a representative. For the synthesis of JC-408, the following amino acids and carboxylic acids were used: (d) Fmoc-L-Phe-OH/DIC/Oxyma; (f) 2-(adamantan-1-yl) acetic acid/DIC/Oxyma. Fmoc: 9-fluorenylmethyloxycarbonyl.

**Synthesis and characterization of 2155-14 and 2155-18.** The desired products were synthesized by solid-phase reactions using an automated microwave-assisted peptide synthesizer, followed by multi-step reactions with conventional oil-bath heating (Scheme 1). Briefly, the CEM Liberty PRIME 2.0 system was used to prepare the key intermediate (**5**) using the one-pot coupling/deprotection methodology. LC analysis of the cleaved samples of the intermediate (**5**) showed one major single absorption at 215 nm, and mass spectrometry analysis showed the matched mass-to-charge ratio (*m*/*z*) with the cleaved (**5**) without the tBu protecting group (m/z calculated for C_40_H_50_N_5_O_6_ [M+H]^+^: 696.38, observed: 696.65). After the structure confirmation of (**5**) by mass and NMR spectrometers, five amide groups were reduced by BH_3_-THF, and boron coordinated with nitrogen of (**6**) was removed by the treatment of piperidine and heating at 65 °C for 18 h. Two 2,3-diketopiperazine units (**7**) were formed by the reaction of (**6**) with 1,1′-oxalyldiimidazole, and the product was cleaved from the resin. The crude product mixture was purified by HPLC, and the final product was characterized by ^1^H and ^13^C NMR (Figure S1A) and high-resolution mass spectrometer (HRMS) (Figure S1B). The other product, 2155-18 (JC-408), was synthesized by following the same method with Fmoc-L-Phe-OH and 2-(adamantan-1-yl) acetic acid in steps d and f in Scheme 1, respectively.

**Figure 1:**
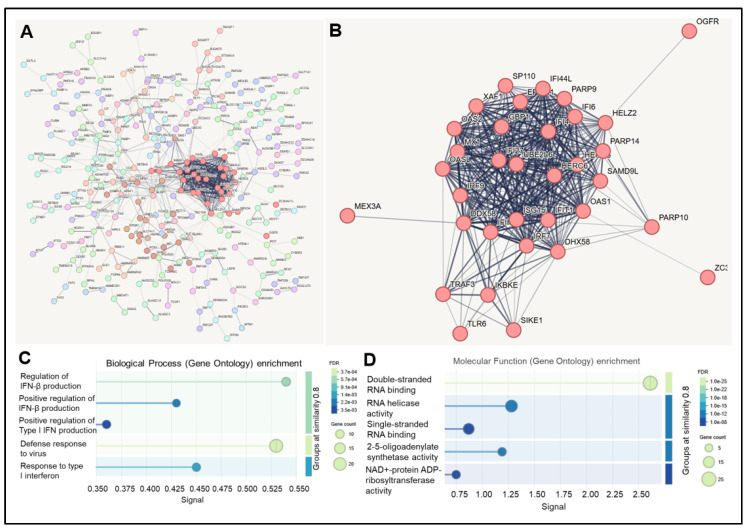
Results of STRING database analysis of DEGs from WM-266-4 melanoma cells treated with hnRNPH2 siRNA. (**A**) DEGs show good protein–protein interactions (PPI enrichment *p*-value: < 1.0 × 10^−16^) and good clustering using the MCL approach (avg. local clustering coefficient: 0.399). (**B**) The largest cluster containing 34 DEGs is enlarged for better visibility. (**C**) Significantly enriched biological processes. (**D**) Significantly enriched molecular functions.

**RNAseq of primary adult melanocytes and the WM-266-4 melanoma cell line.** To obtain insight into the role of hnRNPH2 in melanoma and melanocyte cells, we performed RNA sequencing using cells treated with siRNA targeting hnRNPH2. Treatment of primary adult melanocytes with siRNA resulted in 91 DEGs (Table S1), which did not demonstrate any biological process or pathway enrichment (Figure S2), with only two small local clusters (Figure S2B,C, *n* = 6 genes). These clusters contain genes that were both up- and downregulated, further suggesting the lack of enrichment. In contrast, WM266-4 treated with hnRNPH2 siRNA had 471 DEGs (Table S1), which we analyzed using the STRING database (Figure 1A). Markov Cluster Algorithm (MCL) functional clustering revealed one main cluster (*n* = 34 genes) (Figure 1B). Overall, there was a significant enrichment of several biological processes and molecular functions. The most enriched biological process was Defense Response to Virus (GO:0051607) (*n* = 21 genes, signal = 0.54, FDR = 0.0062) (Figure 1C). The most enriched molecular function was double-stranded RNA (dsRNA) binding (GO:0003725) (*n* = 6 genes, signal = 2.6, FDR = 2.58 × 10^−0.5^) (Figure 1D).

**Figure 2:**
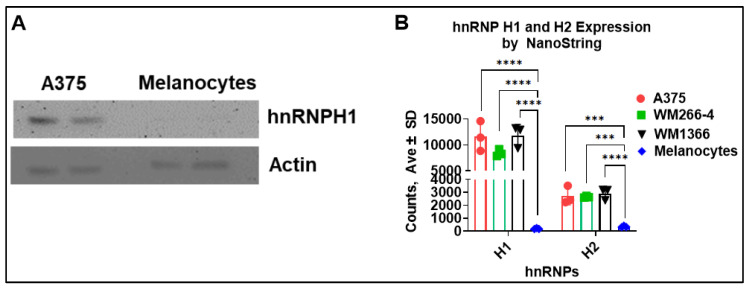
Expression of hnRNPH1 protein in A375 cells and melanocytes and H1 and H2 mRNA in A375, melanocytes, WM1366, and WM266-4 cells. (**A**) Protein expression of H1 in A375 and melanocytes by Western blot (*n* = 2). The original WB images are shown in Figure S5. The statistical significance of differences could not be evaluated due to low expression of H1 in melanocytes. (**B**) Gene expression of hnRNPH1 and H2 in A375, melanocytes, WM1366, and WM266-4 by nCounter (NanoString Inc, Seattle, WA, USA). One-way analysis of variance (ANOVA) was used, followed by the Dunnett post hoc test. The data shown are the mean ± SD, *n* = 3. ***—*p*-value < 0.005, ****—*p*-value < 0.001.

These data suggested that the downregulation of hnRNPH2 expression leads to interferon cell signaling in melanoma but not melanocytes due to possible RNA binding/RNA helicase and oligoadenylate synthetase (OAS) activity. Previously published Western blot analysis has reported that hnRNPH2 protein is not detectable in melanocytes but is readily expressed in WM266-4 [7]. Similarly, H1 protein is not detectable in melanocytes by Western blot (Figure 2A). To further investigate the correlation between hnRNPH1 and hnRNPH2 expression and melanoma, we compared their expression levels in melanoma cell lines and melanocytes from NanoString gene expression analysis. The results revealed that hnRNPH1 and hnRNPH2 gene expression levels were significantly lower in melanocytes compared to melanoma cell lines (Figure 2B). These findings suggest that the lack of effect of hnRNPH2 siRNA on melanocytes is due to the low expression of hnRNPH2 mRNA and protein. Additionally, it is also possible that the hnRNPH2 in melanoma cells interacts with a different repertoire of client pre-mRNAs than in melanocytes.

**Melanoma Cell Lines Following hnRNPH2 siRNA-mediated knockdown.** To determine whether IFN signaling induction by hnRNPH2 siRNA treatment is also observed in other melanoma cell lines, we also included A375, WM266-4 (BRAF-mutant), WM1366 (NRAS-mutant), and WM3918 (triple-wild-type, TWT) in our experiments. Gene expression profiling was performed using nCounter (NanoString)’s Host Immune Response curated panel covering a range of 785 genes from multiple immune signaling pathways [19]. Rosalind v3.16 software was utilized to analyze NanoString data and interpret the large number of DEGs.

hnRNPH2-selective siRNA had little or no effect on hnRNPH1 expression in all three melanoma cell lines (Table 1), with only WM266-4 having a statistically significant difference between the siRNA treatment and scrRNA control groups. The effect on hnRNPH2 expression was significantly greater in melanoma cell lines, suggesting that the downstream effects of knockdown are mostly due to hnRNPH2 expression changes.

**Figure 3:**
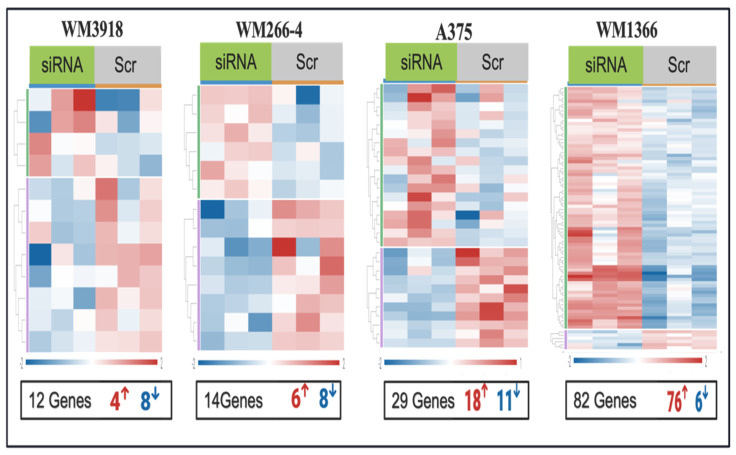
Heatmap clustering of hnRNPH2 siRNA-treated cell lines compared to scrambled control (siRNA vs. Scr). Differentially expressed genes (DEGs) are shown, with upregulated genes in red and downregulated genes in blue, based on the Host Response Panel. The lists of DEGs are available in the Supplementary Material files.

Knockdown of hnRNPH2 using siRNA produced comparable effects in BRAF-mutant cell lines, with A375 exhibiting 29 DEGs (18 upregulated, 11 downregulated genes, Table S2) and WM266-4 showing 14 DEGs (6 upregulated, 8 downregulated genes, Table S3). Remarkably, hnRNPH2 knockdown in WM1366 (NRAS mutated) resulted in the highest number of DEGs, totaling 82 (Table S4). In contrast, WM3918 (TWT) demonstrated the lowest DEGs count, with only 12 genes affected (4 upregulated and 8 downregulated genes, Table S5). Heatmap analysis of the four melanoma cell lines demonstrated distinct clustering of gene expression profiles between treated and control samples (Figure 3).

**Figure 4:**
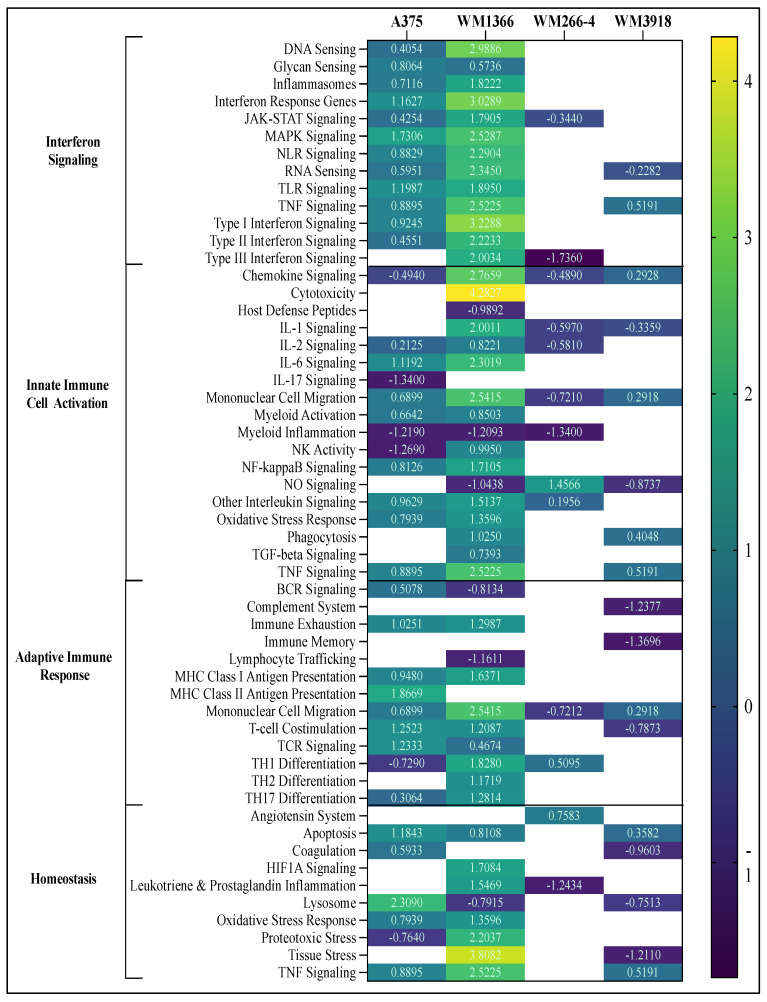
NanoString-based annotation of immune signaling pathways in response to hnRNPH2 siRNA treatment across melanoma cell lines. All values represent DGSSs. Positive values indicate pathway upregulation, and negative values indicate downregulation.

To investigate the biological pathways impacted by hnRNPH2 siRNA treatment, DEGs were annotated using the NanoString nCounter analysis system. Directed global significance scores (DGSSs) were calculated to assess differential expression’s overall strength and direction within key biological processes. The processes were categorized into the five primary functions of the host immune response, including host susceptibility, interferon response, innate immune cell activation, adaptive immune response, and homeostasis (Figure S3). Pathway enrichment analysis was conducted using Rosalind v3.16 software to identify significantly altered cellular processes. Directed global enrichment analysis revealed a strong activation of inflammation-associated pathways following hnRNPH2 siRNA treatment. Among the melanoma cell lines, WM1366 cells exhibited the highest scores across multiple inflammation-associated pathways, particularly those associated with interferon signaling, cytotoxicity, chemokine signaling, tissue stress response, and TNF signaling. hnRNPH2 siRNA treatment of WM266-4 (14 DEGs) and WM3918 (12 DEGs) cell lines had a minimal effect, and the affected pathways were mostly downregulated (Figure 4). In comparison, RNAseq demonstrated 471 DEGs. This difference can be due to the limited gene line-up of the host response panel used for nCounter assay as opposed to the comprehensive scope of the RNAseq approach. A375 (BRAF-mutant) and WM1366 displayed upregulation of Interferon Signaling, while WM266-4 (BRAF-mutant) and WM3918 (TWT) exhibited limited effects. WM1366 had overall greater upregulation of the Interferon Signaling phase compared to A375, with DNA Sensing, Interferon Response Genes, and Type I Interferon Signaling pathways being the most upregulated, whereas the MAPK signaling pathway was the most upregulated in A375 cells (Figure 4). In the Innate Immune Activation phase, A375 response was muted compared to WM1366. A375 displayed the greatest downregulation of chemokine and IL-17 signaling, myeloid inflammation, and NK activity, and upregulation of IL-6, NF-κB, and TNF signaling. WM1366 showed the greatest upregulation of cytotoxicity, IL-6, mononuclear cell migration, and TNF signaling pathways. The adaptive immune response phase was similarly upregulated in both A375 and WM1366 cells. Upregulation of MHC class I and II antigen presentation, T cell co-stimulation, and mononuclear cell migration was the most noteworthy in this phase. In the Homeostasis phase, A375 displayed strong upregulation of lysosomal, apoptotic, and TNF signaling, while WM1366 exhibited proteotoxic and tissue stress and TNF signaling.

**Host Immune Response Following Treatment with 2155-14 and 2155-18.** Next, we treated melanoma and melanocyte cells with 2155-14 and 2155-18 to gain insight into the effect of spliceosomal binding by 2155-14 and 2155-18 on immune signaling and compare it to the effects of siRNA-mediated knockdown of H2. To select the optimal concentration of either compound for these assays, we re-assessed their effect on the viability of melanoma cell lines. We used CellTiter-Glo viability assay and A375, WM266-4, WM1366, WM3918, and melanocytes. Cells were treated with both compounds at final concentrations of 100, 33.33, 11.11, 3.70, 1.23, 0.41, 0.13, 0.04, 0.01, and 0.005 µM for 72 h to assess dose-dependent effects on viability and calculate IC_50_ values.

Compared to the untreated control, both compounds demonstrated dose-dependent cytotoxicity across most melanoma cell lines. Compound 2155-14 significantly reduced the viability of A375 and WM266-4 (BRAF-mutant) and WM1366 (NRAS-mutant) cells, whereas 2155-18 exhibited greater potency against WM266-4 (Table 2 and Figure S4A,B). For WM3918, the viability curve did not reach 50%, thus making the IC_50_ unassignable (Figure S4C). In the melanocyte viability assay, neither compound reduced viability appreciably (Figure S4D).

Since we did not observe any effect on the viability of WM3918 and melanocytes, we focused on A375 and WM1366 cells for further studies. RNA was extracted from three independently treated and untreated cell batches after 8 and 24 h of treatment to capture early and intermediate gene expression responses.

**Figure 5:**
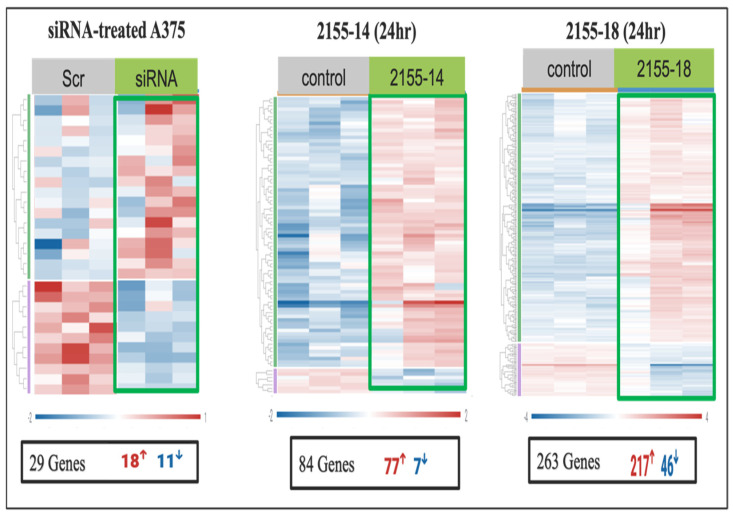
Heatmap clustering of hnRNPH2 siRNA-treated A375 vs. compound-treated A375 cells. Differentially expressed genes (DEGs) are shown, with upregulated genes in red and downregulated genes in blue, based on the Host Response Panel.

**Figure 6:**
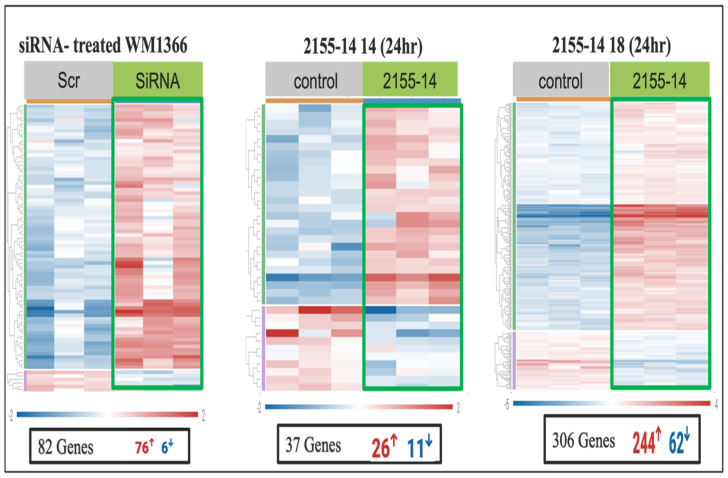
Heatmap clustering of hnRNPH2 siRNA-treated vs. compound-treated WM1366 cells. Differentially expressed genes are shown, with upregulated genes in red and downregulated genes in blue, based on the Host Response Panel.

NanoString analysis revealed time-dependent changes in transcription response to either 2155-14 or 2155-18 treatments, with more pronounced gene expression changes observed at 24 h compared to those observed at 8 h. Due to the low gene expression changes after 8 h of treatment, subsequent analyses focus on the 24 h data. Treatment of A375 cells with 2155-14 resulted in 84 DEGs (77 upregulated, 7 downregulated genes) (Supplementary Table S6). In contrast, 2155-18 had a more pronounced effect on A375, leading to 263 DEGs (217 upregulated, 46 downregulated genes) (Supplementary Table S7). In WM1366 cells, 2155-14 treatment resulted in 37 DEGs (26 upregulated, 11 downregulated genes) (Supplementary Table S8). Meanwhile, treatment with 2155-18 induced 306 DEGs, with 244 upregulated and 62 downregulated genes (Supplementary Table S9). Heat map analysis demonstrated distinct clustering of gene expression profiles between treated and control samples, highlighting clear transcriptional alterations induced by each compound. The heatmap displayed an upper cluster dominated by upregulated genes in treated samples, whereas genes in the lower cluster were predominantly downregulated. However, the degree of differential expression varied between the cell lines and compounds, with 2155-18 producing greater gene expression perturbation as compared to 2155-14 and siRNA (Figure 5 and Figure 6).

**Figure 7:**
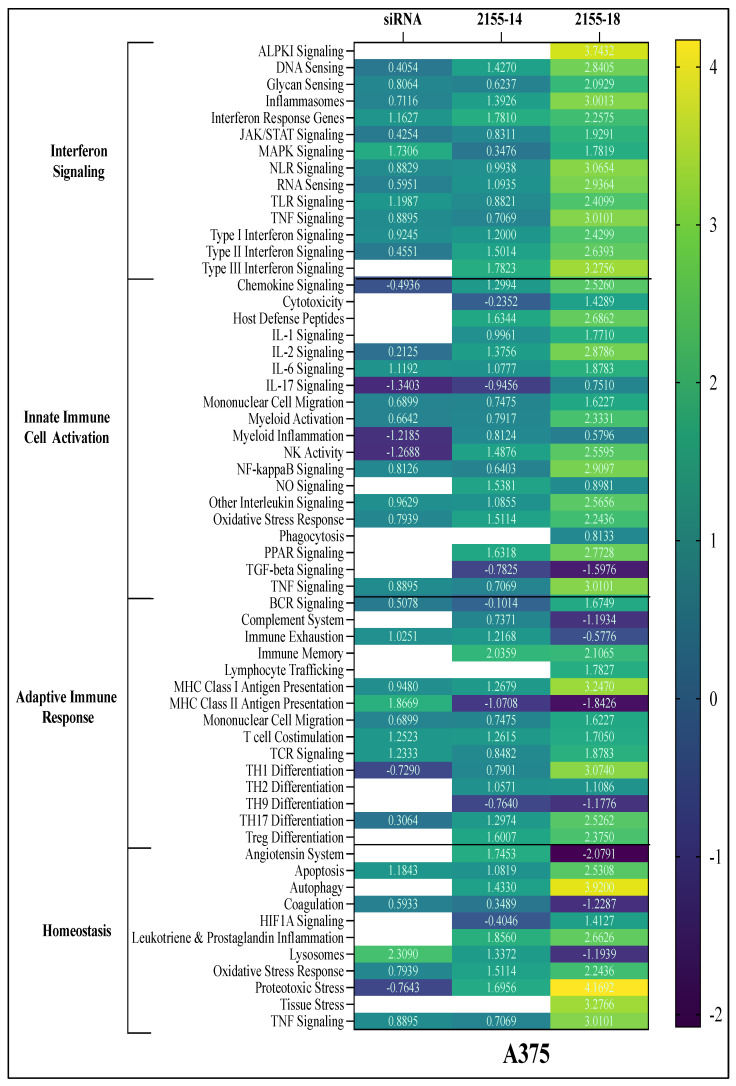
NanoString-based annotation of immune signaling pathways in response to hnRNPH2 siRNA, 2155-14 and 2155-18 treatment of A375. All values represent DGSSs.

Directed global enrichment analysis in A375 melanoma cells revealed that treatment with 2155-14 and 2155-18 activated more signaling pathways compared to hnRNPH2 siRNA treatment. Among the two compounds, 2155-18 activated more pathways compared to 2155-14. The top enriched pathways included interferon-related pathways, multiple interleukin pathways (IL-1, IL-2, IL-6, IL-17), and inflammasome activation. Additionally, MHC Class I antigen presentation, TNF signaling, proteotoxic stress, tissue stress, and autophagy were significantly upregulated following compound treatment. In contrast, TGFβ signaling was notably downregulated (Figure 7).

**Figure 8:**
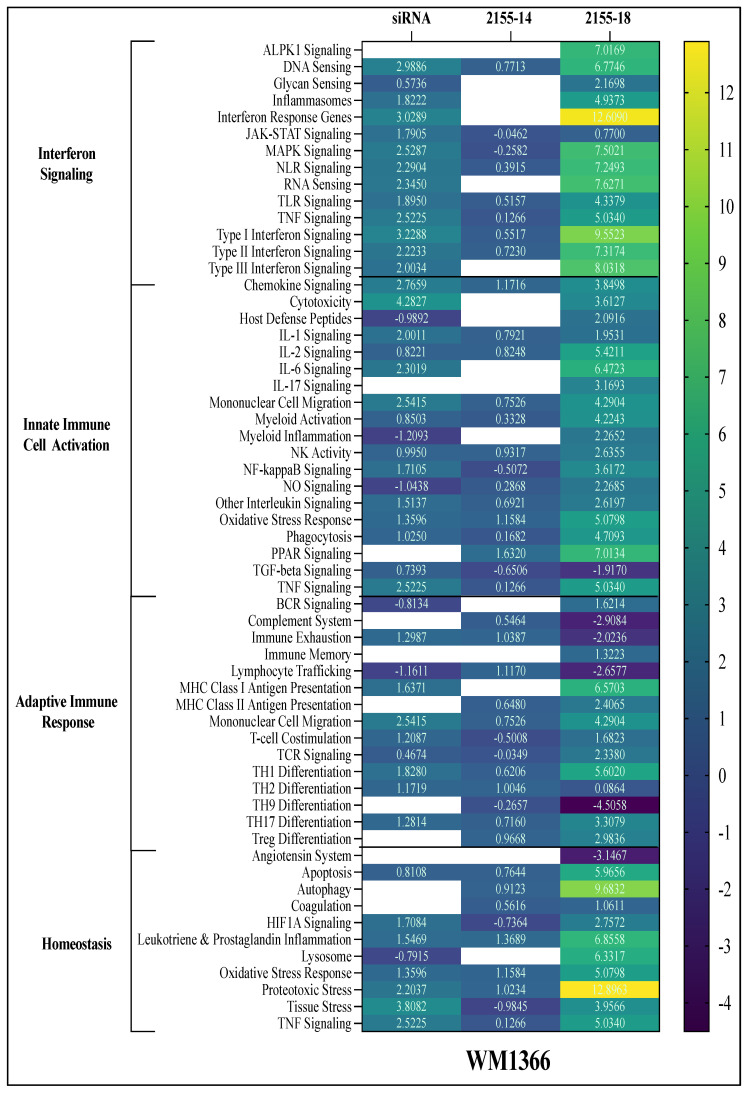
NanoString-based annotation for immune signaling pathways in response to hnRNPH2 siRNA, 2155-14 and 2155-18 treatment of WM1366. All values represent DGSSs.

Similarly, directed global enrichment analysis in WM1366 cells showed that both compounds modulated immune and stress response pathways, though the magnitude and pattern of activation differed from A375. Notably, 2155-18 treatment induced a stronger upregulation of interferon response, TNF signaling, and antigen presentation pathways. Inflammasome activation and IL signaling (IL-1, IL-2, IL-6, IL-6, IL-17) were also enriched. Unlike A375, WM1366 exhibited a pronounced upregulation of oxidative and proteotoxic stress. TGF-beta signaling was also downregulated (Figure 8).

## 4. Discussion

The results presented in this study demonstrate the impact of 2155-14, 2155-18, and hnRNPH2 siRNA in downregulating spliceosomal proteins hnRNPH1 and hnRNPH2 in melanoma cells. We also demonstrate that both genetic and pharmacological downregulation of the two proteins led to significant upregulation of pro-inflammatory signaling pathways, particularly those related to antigen presentation, interferon signaling pathways, interleukin regulation, apoptosis, and autophagy. Conversely, there was notable downregulation of anti-inflammatory pathways, most prominently the TGF-β signaling pathway.

The viability assays in the present study revealed differential effects of compounds 2155-14 and 2155-18 on cell viability across various melanoma cell lines, with no significant impact on cell survival observed in normal primary melanocytes. We reported previously that neither compound affects other non-malignant cell types, including those from the kidney, ovary, and brain [7]. In addition, we demonstrated no impact on several non-melanoma cancer cell lines, such as those derived from lung, liver, and brain cancers. This selective activity highlights both a potentially safe therapeutic window and a melanoma-specific effect, which are critical for future clinical applications.

The NanoString assays revealed significantly lower gene expression levels of hnRNPH1 and hnRNPH2 in untreated melanocytes compared to untreated melanoma cell lines, consistent with our group’s previous Western blot findings [7]. To our knowledge, it has not been demonstrated previously, as hnRNPH1 and hnRNPH2 are believed to be expressed ubiquitously. Members of the hnRNP protein family have been implicated in various aspects of tumor development and progression, including the promotion of cell proliferation, migration, and inflammatory signaling [20,21,22]. Furthermore, our group demonstrated that treating WM266-4 with 2155-14 led to a reduction in hnRNPH1 and hnRNPH2 protein levels [7]. Notably, our NanoString gene expression data from WM266-4 cells treated with 2155-14 did not show a corresponding decrease in hnRNPH1 and hnRNPH2 mRNA levels. This discrepancy between protein and mRNA levels suggests that the mechanism of action of 2155-14 may occur at the post-transcriptional level, potentially through inhibition of translation or by destabilizing the hnRNPH2 protein [23].

Among the most enriched responses following hnRNPH2 siRNA treatment were those linked to interferon signaling, cytotoxicity, chemokine signaling, tissue stress, and TNF signaling. These pathways are well known to stimulate anti-tumor immunity, inflammation, and immune surveillance, and their upregulation may reflect heightened immune responsiveness in the treated cell lines [24]. Activation of chemokine signaling suggests migrating immune cells, including NK cells, DCs, and CD8^+^ T cells, into the tumor microenvironment [25]. Following treatment of A375 and WM1366 cells with 2155-14 and 2155-18, several immune-related pathways were significantly enriched. This included interferon signaling, multiple interleukin pathways (IL-2, IL-6, IL-17), antigen presentation via MHC I, TNF signaling, PPAR signaling, and inflammasome activation. In addition to these immune pathways, we observed upregulation of stress response mechanisms such as proteotoxic stress, oxidative stress responses, apoptosis, and autophagy. These findings are consistent with previous results from our group. Specifically, our group’s prior studies demonstrated that 2155-14 treatment led to a significant increase in late-stage apoptosis and the induction of autophagy markers in melanoma cells. Moreover, our group also reported that 2155-14 induces endoplasmic reticulum (ER) stress in melanoma, a critical component of proteotoxic stress responses [7].

Host immune response evaluation revealed notable differences in the magnitude of immune activation between hnRNPH2 siRNA treatment and pharmacological compounds 2155-14 and 2155-18. The broader activation of immune-related signaling pathways observed with compound treatments, as opposed to the genetic modulation of hnRNPH2, may be attributed to several factors, such as possible polypharmacology of 2155-14 and 2155-18. We previously reported that the biotinylated version of 2155-14 pulled down several proteins (hnRNPH2 and, possibly, H1, hnRNP A2/B1, and DDX1) with relevance in pre-mRNA splicing [7]. The pharmacological effects of 2155-14 and 2155-18 treatment may be due to the binding to all these proteins (i.e., polypharmacology), which would explain the greater impact on immune signaling as compared to siRNA treatment. This result certainly warrants further exploration. Additionally, hnRNPH2 siRNA specifically targets and depletes a single mRNA, resulting in a more focused and limited downstream effect. In contrast, the small-molecule compounds may interact with multiple cellular targets, including potential unknown or off-target molecules, thereby amplifying their downstream immunomodulatory effects.

It is also worth mentioning that the observed downregulation of several immune-activating pathways likely reflects a regulatory mechanism to prevent overactivation and maintain immune equilibrium. The immune system maintains homeostasis by regulating the interplay between activating signals that promote immune responses and inhibitory mechanisms that prevent excessive or aberrant activation. This balance is crucial for proper immune function and avoiding excessive inflammatory responses [26]. Our future studies will focus on melanoma patient cell cultures carrying different mutational backgrounds.

## 5. Conclusions

The results of this study offer valuable insights into pharmacologic and siRNA targeting of hnRNPH1 and H2 proteins in melanoma cells with respect to their immunomodulatory effects in melanoma. To our knowledge, this is the first study to investigate the roles of hnRNPH2 and other RNA-binding proteins (RBPs) in melanoma immune signaling. The observed effects from targeting this protein suggest that it represents a novel molecular driver of melanoma immune response and may open avenues for therapeutic development.

## Figures and Tables

**Table 1:** Results of nCounter analysis of hnRNPH1 and hnRNPH2 expression after siRNA treatment in melanoma cells and primary adult melanocytes. All data are averages, *n* = 3. Statistical comparisons between two means were conducted using an unpaired Student’s *t*-test.

Target	Cells/Treatment	scRNA Control, Counts	siRNA Treated, Counts	Fold Change, Counts	*p*-Value
hnRNPH1	A375 + siRNA	10,121.21	8564.34	−1.18	0.15282391
	WM266-4 + siRNA	8134.36	5576.84	−1.46	0.03156231
	WM1366 + siRNA	11,967	10,602.6	−1.13	0.07347471
	Melanocytes + siRNA	237.67	277.15	1.17	0.16522355
hnRNPH2	A375 + siRNA	2365.29	444.09	−5.33	0.00030667
	WM266-4 + siRNA	2553.57	109.41	−23.34	0.00035173
	WM1366 + siRNA	2958.53	310.89	−9.52	0.00440947
	Melanocytes + siRNA	317.79	173.88	−1.83	0.02992843

**Table 2:** Viability assay results of 2155-14 and 2155-18 in melanoma and melanocyte cells. All results are presented as IC_50_ values (µM), Ave ± SD (*n* = 3). WM266-4—^V600D^BRAF/^−/−^PTEN, A375—^V600G^BRAF/CDKN2A^D61T^, WM1366—NRAS^Q61L^/TP53^D285K^, WM3918—CDKN2A^−/−^. All cell line information was obtained from cellosaurus.org.

Compound	A375	WM266-4	WM1366	WM3918	Melanocytes
2155-14	16.6 ± 1.8	11.3 ± 1.2	15.5 ± 1.2	>100	>100
2155-18	29.5 ± 3.0	9.63 ± 1.0	77.3 ± 15.4	>100	>100

## Data Availability

The original contributions presented in this study are included in the article/Supplementary Materials. Further inquiries can be directed to the corresponding author.

## Supplementary Materials

The following supporting information can be downloaded at https://www.mdpi.com/article/10.3390/biom15111611/s1. Figure S1: (A) 1H and 13C NMR spectra of JC-395. (B) High-resolution mass spectrometry analysis of JC-395. Figure S2: String database analysis of primary adult melanocytes treated with hnRNPH2 siRNA. Figure S3: Pathway annotations across the five functions of the host response. Figure S4: Viability assay results of 2155-14 and 2155-18 in melanoma and melanocyte cells. Figure S5: Original Western blot images of hnRNPH1 and Actin in A375, melanocytes, and WM266-4 cells. Table S1: DEGs from RNAseq analysis of mRNAs from WM-266-4 and melanocyte cells treated with hnRNPH2 siRNA. Table S2: Results of nCounter analysis of mRNAs from A375 cells treated with hnRNPH2 siRNA using the Host Response Panel. Only DEGs are listed out of 785 genes the RNAs were analyzed against. Table S3: Results of nCounter analysis of mRNAs from WM-266-4 cells treated with hnRNPH2 siRNA using the Host Response Panel. Only DEGs are listed out of 785 genes the RNAs were analyzed against. Table S4: Results of nCounter analysis of mRNAs from WM1366 cells treated with hnRNPH2 siRNA using the Host Response Panel. Only DEGs are listed out of 785 genes the RNAs were analyzed against. Table S5: Results of nCounter analysis of mRNAs from WM3918 cells treated with hnRNPH2 siRNA using the Host Response Panel. Only DEGs are listed out of 785 genes the RNAs were analyzed against. Table S6: Results of nCounter analysis of mRNAs from A375 cells treated with 2155-14 using the Host Response Panel. Only DEGs are listed out of 785 genes the RNAs were analyzed against. Table S7: Results of nCounter analysis of mRNAs from A375 cells treated with 2155-18 using the Host Response Panel. Only DEGs are listed out of 785 genes the RNAs were analyzed against. Table S8: Results of nCounter analysis of mRNAs from WM1366 cells treated with 2155-14 using the Host Response Panel. Only DEGs are listed out of 785 genes the RNAs were analyzed against. Table S9: Results of nCounter analysis of mRNAs from WM1366 cells treated with 2155-18 using the Host Response Panel. Only DEGs are listed out of 785 genes the RNAs were analyzed against.
