# Supplementary material for: Modulation of Spliceosomal Proteins hnRNPH1 and H2 Increases Melanoma Cell Pro-Inflammatory Signaling In Vitro
Source: Biomolecules. 2025 Nov 17;15(11):1611. doi: 10.3390/biom15111611 (PMC12649858; doi:10.3390/biom15111611)
Supplement: Supplementary file 1 [file biomolecules-15-01611-s001.zip › Supplementary Figures rev S1.pdf]

biomolecules

MDPI

Supplementary Materials

Modulation of Spliceosomal Proteins hnRNPH1 and H2 Increases Melanoma Cell Pro-Inflammatory Signaling In Vitro

Maab Sultan [1], Shuai Ma[2], Juan Diez [1,3], Sadeeshkumar Velayutham [1,3,], Yousef Al-Harbi [1,4], Jun Yong Choi [2,5], Keiran S.M. Smalley [6], Lubov Nathanson [7], Vladimir Beljanski [8], and Dmitriy Minond [1,3]

[1] Barry and Judy Silverman College of Pharmacy, Nova Southeastern University, 3321 College Avenue, Fort Lauderdale, FL 33314.

[2] Ph.D. Program in Chemistry, The Graduate Center of the City University of New York, New York, NY, 10016

[3] Rumbaugh-Goodwin Institute for Cancer Research, Nova Southeastern University, 3321 College Avenue, CCR r.605, Fort Lauderdale, FL 33314.

[4] Department of Pharmacology and Toxicology, College of Pharmacy, Qassim University, Buraidah, Saudi Arabia

[5] Department of Chemistry and Biochemistry, Queens College, 65-30 Kissena Boulevard, Flushing, NY 11367

[6] Department of Tumor Biology, Moffitt Cancer Center, 12902 Magnolia Drive, Tampa, FL 33612.

[7] Dr. Kiran C. Patel College of Osteopathic Medicine, Nova Southeastern University, 3321 College Avenue, Fort Lauderdale, FL 33314.

[8] Dr. Kiran C. Patel College of Allopathic Medicine, Nova Southeastern University, 3321 College Avenue, Fort Lauderdale, FL 33314.

Academic Editor: Firstname Lastname

Received: date
Revised: date
Accepted: date
Published: date

Citation: To be added by editorial staff during production.

Copyright: © 2025 by the authors. Submitted for possible open access publication under the terms and conditions of the Creative Commons Attribution (CC BY) license (https://creativecommons.org/licenses/by/4.0/).

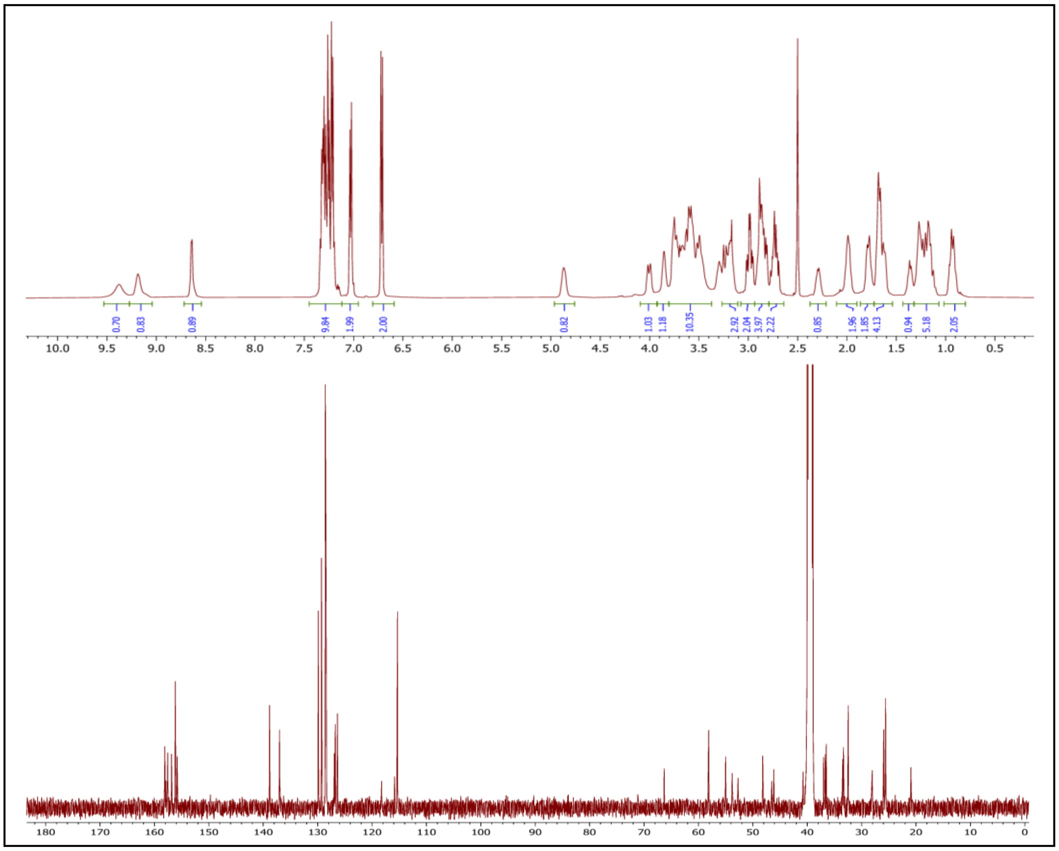

Supplementary Figure S1A. 1H and 13C NMR spectra of JC-395.

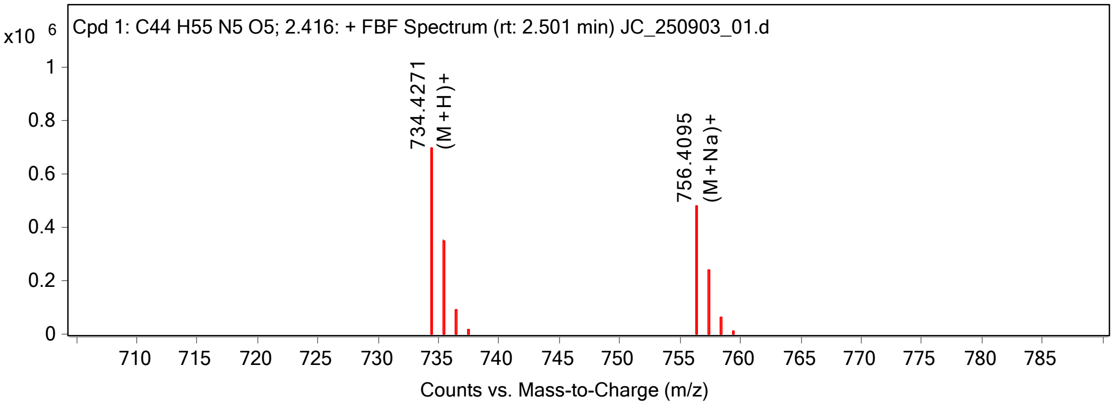

Supplementary Figure S1B. High resolution mass spectrometry analysis of JC-395.

biomolecules

MDPI

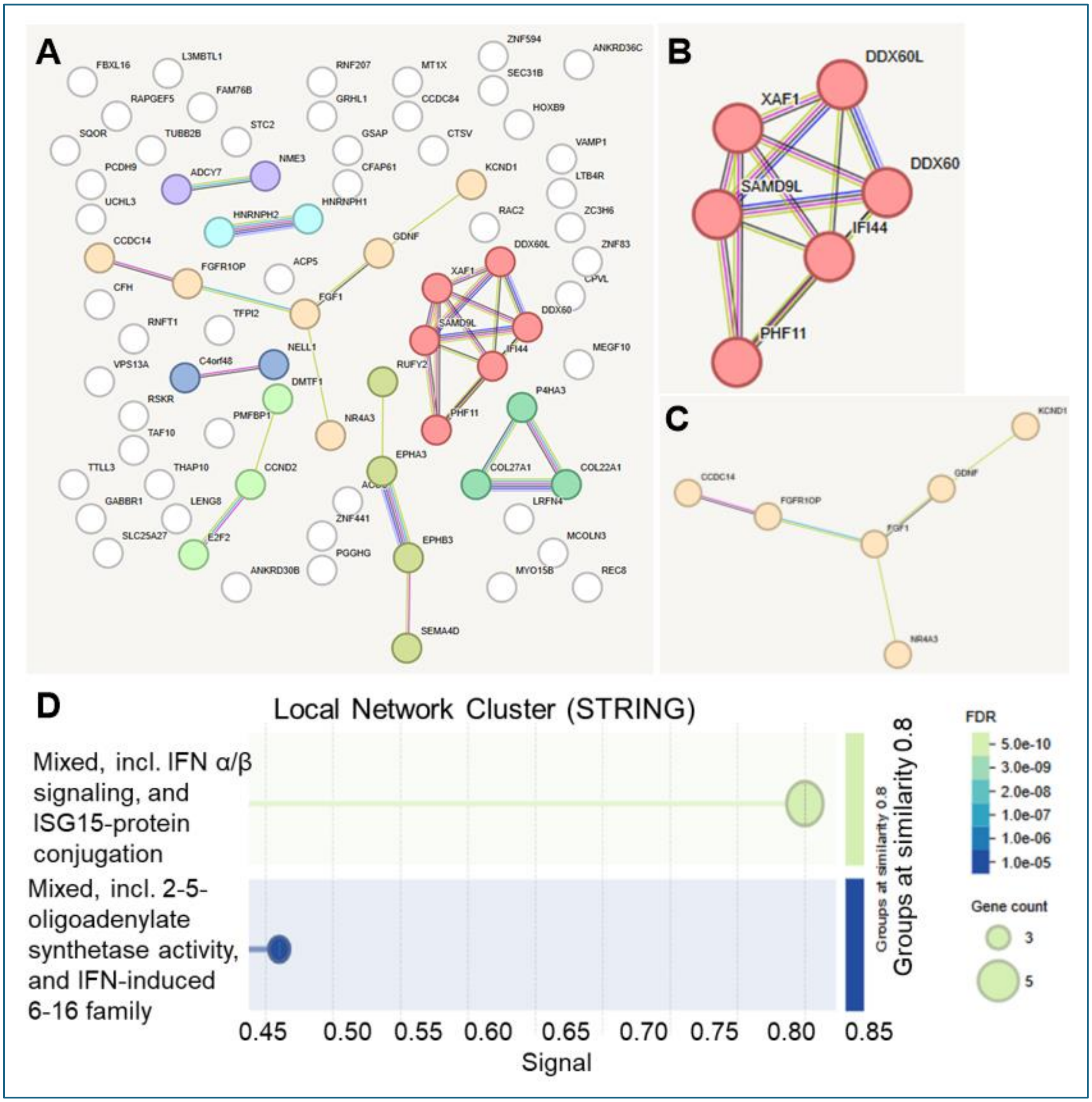

Supplementary Figure S2. String database analysis of primary adult melanocytes treated with hnRNPH2 siRNA. (A) Visualization of 91 DEGs shows neither process nor pathway enrichment, with only two small local clusters (B and C, n=6 genes) (D) Local clusters analysis of (B).

biomolecules

MDPI

Host Susceptibility (26 Genes Human/ 15 Genes Mouse)	Interferon Response (288 Genes Human/ 230 Genes Mouse)	Innate Immune Cell Activation (567 Genes Human/ 383 Genes Mouse)	Adaptive Immune Response (483 Genes Human/ 439 Genes Mouse)	Homeostasis (282 Genes Human/ 220 Mouse)
Angiotensin System	ALPK1 Signaling	Chemokine Signaling	BCR Signaling	Angiotensin System
Virus-Host Interaction	DNA Sensing	Cytotoxicity	Complement System	Apoptosis
	Glycan Sensing	Host Defense Peptides	Immune Exhaustion	Autophagy
	Inflammasomes	IL-1 Signaling	Immune Memory	Coagulation
	Interferon Response Genes	IL-2 Signaling	Lymphocyte Trafficking	HIF1A Signaling
	JAK/STAT Signaling	IL-6 Signaling	MHC Class I Antigen Presentation	Leukotriene and Prostaglandin Inflammation
	MAPK Signaling	IL-17 Signaling	MHC Class II Antigen Presentation	Lysosomes
	NLR Signaling	Mononuclear Cell Migration	Mononuclear Cell Migration	Oxidative Stress Response
	RNA Sensing	Myeloid Activation	T cell Costimulation	Proteotoxic Stress
	TLR Signaling	Myeloid Inflammation	TCR Signaling	Tissue Stress
	TNF Signaling	NK Activity	TH1 Differentiation	TNF Signaling
	Type I Interferon Signaling	NF-kappaB Signaling	TH2 Differentiation	
	Type II Interferon Signaling	NO Signaling	TH9 Differentiation	
	Type III Interferon Signaling	Other Interleukin Signaling	TH17 Differentiation	
		Oxidative Stress Response	Treg Differentiation	
		Phagocytosis		
		PPAR Signaling		
		TGF-beta Signaling		
		TNF Signaling		

Supplementary Figure S3. Pathway annotations across the five functions of the host response. **Reproduced From:** [19]

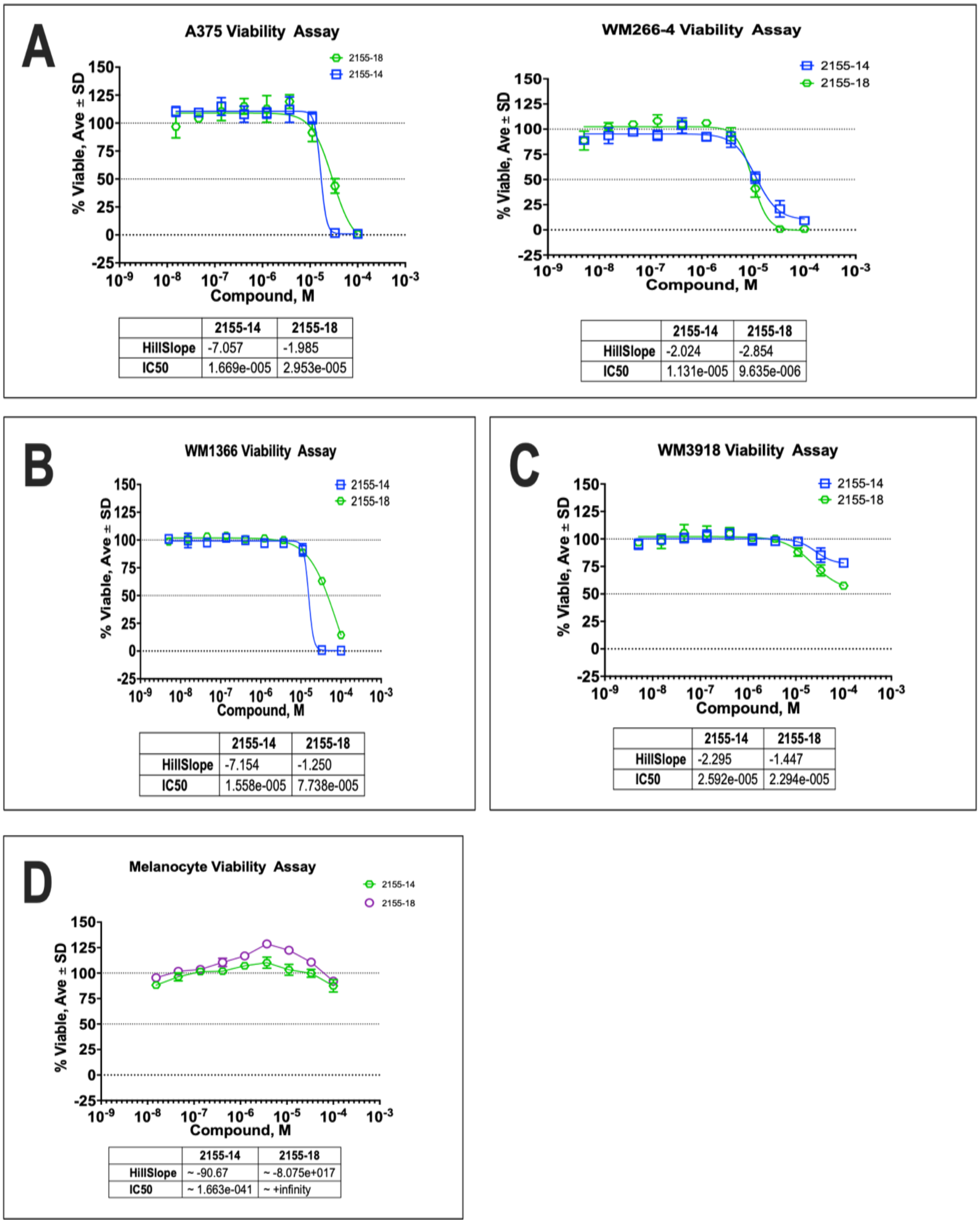

A375

	2155-14	2155-18
HillSlope	-7.057	-1.985
IC50	1.669e-005	2.953e-005

WM266-4

	2155-14	2155-18
HillSlope	-2.024	-2.854
IC50	1.131e-005	9.635e-006

WM1366

	2155-14	2155-18
HillSlope	-7.154	-1.250
IC50	1.558e-005	7.738e-005

WM3918

	2155-14	2155-18
HillSlope	-2.295	-1.447
IC50	2.592e-005	2.294e-005

Melanocyte

	2155-14	2155-18
HillSlope	~ -90.67	~ -8.075e+017
IC50	~ 1.663e-041	~ +infinity

Supplementary Figure S4. Viability assay results of 2155-14 and 2155-18 in melanoma (A-C) and melanocyte (D) cells.

biomolecules

MDPI

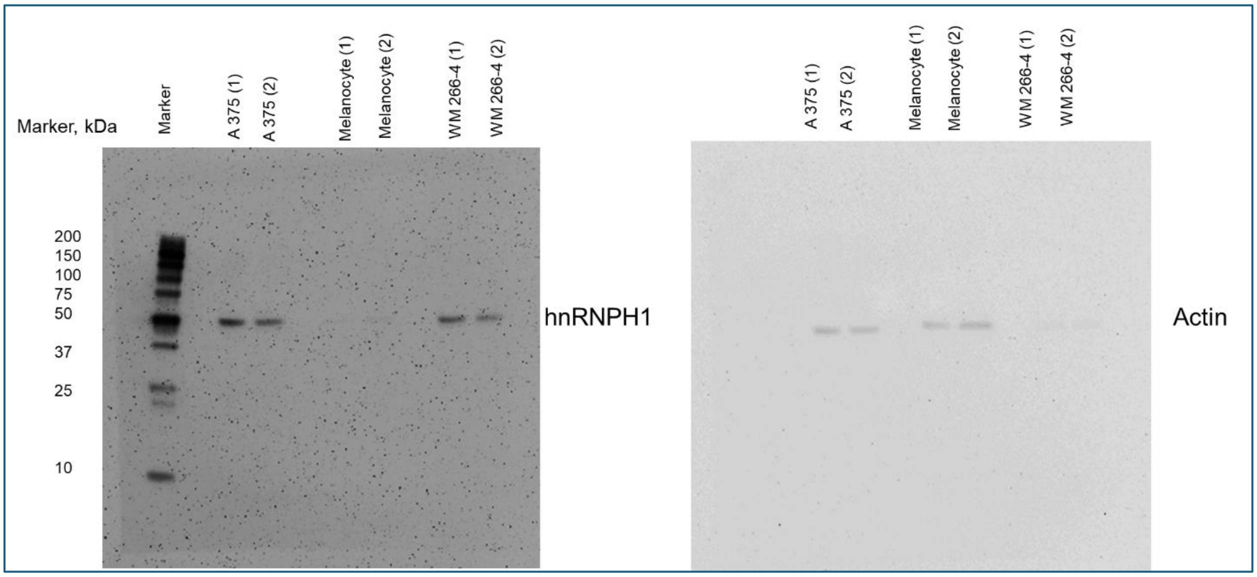

Supplementary Figure S5. Original western blot images of hnRNPH1 and Actin in A375, melanocytes, and WM266-4 cells.
